# Predicting the survival of patients with bone metastases treated with radiation therapy: a validation study of the Katagiri scoring system

**DOI:** 10.1186/s13014-019-1218-z

**Published:** 2019-01-18

**Authors:** Hikaru Kubota, Toshinori Soejima, Nor Shazrina Sulaiman, Shuhei Sekii, Yoko Matsumoto, Yosuke Ota, Kayoko Tsujino, Ikuo Fujita, Takuya Fujimoto, Masayuki Morishita, Junichi Ikegaki, Koji Matsumoto, Ryohei Sasaki

**Affiliations:** 1 Department of Radiation Oncology, Hyogo Cancer Center, 13-70, Kita-oji, Akashi, Hyogo Japan; 2 Department of Radiation Oncology, Kobe University Hospital, 7-5-2 Kusunoki-cho, Chuo-ku, Kobe, Hyogo Japan; 3 Department of Radiation Oncology, Kobe Proton Center, 1-6-8, Minatojima-minami-cho, Chuo-ku, Kobe, Japan; 4 Department of Orthopaedic Surgery, Hyogo Cancer Center, 13-70, Kita-oji, Akashi, Hyogo Japan; 5 Department of Palliative Medicine, Hyogo Cancer Center, 13-70, Kita-oji, Akashi, Hyogo Japan; 6 Department of Medical Oncology, Hyogo Cancer Center, 13-70, Kita-oji, Akashi, Hyogo Japan

**Keywords:** Bone metastasis, Prognostic factors, Katagiri scoring system, Palliative radiation therapy, Optimizing dose-fractionation

## Abstract

**Background:**

The selection of radiation therapy dose fractionation schedules for bone metastases is often based on the estimation of life expectancy. Therefore, accurate prognosis prediction is an important issue. It is reported that the Katagiri scoring system can be used to predict the survival of patients with bone metastases. We aimed to assess prognostic factors and validate the Katagiri scoring system in patients who were treated with radiation therapy for bone metastases.

**Materials/Methods:**

We retrospectively reviewed data of all patients who were treated with radiation therapy for bone metastases between 2004 and 2013. Age, sex, Karnofsky performance status (KPS), Eastern Cooperative Oncology Group performance status (ECOG PS), primary site (lesions and characteristics), visceral metastases, laboratory data, previous chemotherapy, and multiple bone metastases were analyzed for associations with overall survival (OS). Katagiri scores were calculated for each patient and were used to compare OS.

**Results:**

Out of the 616 patients included in this analysis, 574 had died and 42 remained alive. The median follow-up time for survivors was 42 months. Univariate analysis revealed that age (*P* = 0.604) and multiple bone metastases (*P* = 0.691) were not significantly associated with OS. Multivariate analysis revealed that sex, ECOG PS, KPS, primary characteristics, visceral metastases, laboratory data, and previous chemotherapy were significantly associated with OS. The survival rates at 3, 6, 12, and 24 months, categorized by Katagiri score, were as follows: score 0–3, 94.4, 77.8, and 61.1%, respectively; score 4–6, 67.7, 48.7, and 31.2%, respectively; and score 7–10, 39.1, 22.1, and 9.0%, respectively (*P* < 0.001).

**Conclusion:**

Sex, ECOG PS, KPS, primary characteristics, visceral metastases, laboratory data, and previous chemotherapy were significant predictors of survival in patients with bone metastases. The Katagiri scoring system was significantly correlated with OS and can help us select the optimal dose-fractionation.

**Electronic supplementary material:**

The online version of this article (10.1186/s13014-019-1218-z) contains supplementary material, which is available to authorized users.

## Background

Radiation therapy (RT) plays important roles in the palliation of symptomatic bone metastases, such as in pain relief, management of metastatic spinal cord compression (MSCC), and treatment of oligometastases. The selection of RT dose-fractionation schedules for bone metastases is often based on the estimation of life expectancy. While the accurate prediction of survival can lead to the selection of optimal dose-fractionation schedules, it is well recognized that clinician predictions of survival are often inaccurate [1]. It is necessary to clarify prognostic factors and establish accurate survival prediction systems for patients with bone metastases.

There have been several reports on the prognosis of patients with bone metastases. The Katagiri scoring system is one prognostic scoring system that is based on a prospective, single center analysis of 808 patients with symptomatic bone metastases. This system comprises the six prognostic factors that were found to be significantly associated with survival in the multivariate analysis of those 808 patients. Though most previous studies regarding prognostic factors for patients with bone metastases have analyzed metastases of the spine alone and patients who were treated with either surgery or RT, the Katagiri scoring system captured metastases of the entire skeleton and analyzed patients who were treated with both surgery and RT [2]. In the Katagiri scoring system, the primary lesion, visceral or cerebral metastases, abnormal laboratory data, poor performance status, previous chemotherapy, and multiple skeletal metastases are selected as significant prognostic factors. The survival curves can be separated into three groups, based on the respective survival rates at 12 months: the low-risk group (score of ≤3), for survival rates > 80% at 12 months; the intermediate-risk group (score of 4–6), for survival rates of 30–80%; and the high-risk group (score of 7–10), for survival rates ≤10% at 12 months.

In this study, we investigated patient survival from the start of RT. Our objectives were to assess prognostic factors in patients who were treated with RT for bone metastases and to validate the Katagiri scoring system with our data.

## Patients and methods

This study was a retrospective investigation of patients who were treated with radiation therapy for bone metastases between 2004 and 2013 at our hospital. Patients were excluded from the study if their bone metastases had been irradiated previously. We analyzed the effects of nine potential prognostic factors on patient survival after radiation therapy for bone metastases, and these patients represented the validation group for the Katagiri scoring system.

The Institutional Review Board (IRB) of our hospital approved this study. In 2017, survival data were obtained from the medical records or the investigation, based on ethical consideration.

### The potential prognostic factors

The potential prognostic factors included age, sex, Karnofsky Performance Status (KPS), Eastern Cooperative Oncology Group Performance Status (ECOG PS), laboratory data, primary site (legion and characteristics), visceral or cerebral metastases, previous chemotherapy, and multiple bone metastases. Primary characteristics, laboratory data, and visceral metastases were referred to the Katagiri scoring system [2]. Sex was classified into two categories: male or female. Age was classified into two categories: ≤64 or ≥ 65 years. KPS was classified into three categories: KPS 0–60, 70–80, and 90–100. ECOG PS was classified into two categories: PS 0–2 and 3–4. The primary tumor site was categorized by lesion identity and characteristics, and primary lesions were classified into four groups: breast, prostate, lung, and others. The primary characteristics were classified into three groups: tumors that exhibited slow growth (breast cancer, prostate cancer, thyroid cancer, multiple myeloma, and malignant lymphoma), moderate growth (renal cell carcinoma, endometrial and ovarian cancer, sarcoma, and others) or rapid growth (lung cancer, colorectal cancer, gastric cancer, pancreatic cancer, head and neck cancer, esophageal cancer, other urological cancers, melanoma, hepatocellular carcinoma, gall bladder cancer, cervical cancer, and cancers of unknown origin). Visceral or cerebral metastases were classified into three categories: no visceral or cerebral metastasis, ordinary nodular metastasis, and disseminated metastasis, such as pleural, peritoneal, or leptomeningeal dissemination. Laboratory data included abnormalities of C-reactive protein (CRP) (≥0.4 mg/dL), lactate dehydrogenase (LDH) (≥250 IU/L), serum albumin (< 3.7 g/dL), platelet count (< 100,000/μL), serum calcium level (≥10.3 mg/dL), or total bilirubin (≥1.4 mg/dL). Laboratory data were classified into three categories: normal, abnormal (CRP, LDH, or serum albumin), and critical (platelet count, serum calcium level, or total bilirubin). Laboratory data were collected until 2 months prior to irradiation, and serum calcium was corrected for albumin level. Previous chemotherapy was classified into two categories: yes and no. The number of bone metastases was classified into two categories: solitary and multiple bone metastases.

### Radiation therapy

RT was performed mainly with 4-, 6- or 10-MV photons using linear accelerators. A three-dimensional treatment-planning system was performed in all patients. Three-dose fractionation schedules were typically feasible in patients with bone metastases and included single-fraction RT, such as 8 Gy in 1 fraction, or multi-fraction RT, such as 20 Gy in 5 fractions and 30 Gy in 10 fractions. The RT schedule was selected mainly based on clinician prognosis predictions and radiotherapy purpose, which was to relieve pain, manage MSCC, prevent pathological fracture, or control oligometastases.

### The Katagiri scoring system

The Katagiri scoring system [2] comprises six prognostic factors, which include the primary characteristics, visceral metastases, laboratory data, ECOG PS, previous chemotherapy, and multiple bone metastases (Table 1). For the primary characteristics category, patients with lung cancer were categorized into two subgroups, based on treatment or not with molecular targeted agents (gefitinib and/or erlotinib), and patients with prostate and breast cancers were categorized according to sensitivity to hormonal therapy, based on the original Katagiri scoring system. Furthermore, we simplified lung cancer categories based on rapid growth tumor, and breast and prostate cancer categories based on slow growth tumor growth, due to the difficulty in retrospectively finding data. In this scoring system, the score for each prognostic factor ranged from 0 to 3. The Katagiri score represented the sum of the scores for each factor and ranged from 0 to 10. Katagiri scores were categorized into three groups as follow: scores of 0–3 were classified as low-risk, 4–6 as intermediate-risk, and 7–10 as high-risk.

**Table 1:** Katagiri’s scoring system

Prognostic factor	Score
Primary characteristics^a^	
Slow^1^	0
Moderate^2^	2
Rapid^3^	3
Visceral metastases	
No	0
Nodular	1
Disseminated^4^	2
Laboratory data	
Normal	0
Abnormal^5^	1
Critical^6^	2
ECOG PS	
0–2	0
3–4	1
Previous chemotherapy	
No	0
Yes	1
Multiple skeletal metastases	
No	0
Yes	1
Total	10

1: Breast cancer, prostate cancer, thyroid cancer, multiple myeloma, and malignant lymphoma; 2:Renal cell carcinoma, endometrial and ovarian cancer, sarcoma, and others; 3: Lung cancer, colorectal cancer, gastric cancer, pancreatic cancer, head and neck cancer, esophageal cancer, other urological cancers, melanoma, hepatocellular carcinoma, gall bladder cancer, cervical cancer, and cancers of unknown origin; 4: Pleural, peritoneal, or leptomeningeal dissemination; 5: CRP ≥ 0.4 mg/dL, LDH ≥ 250 IU/L, or serum albumin < 3.7 g/dL; 6: platelet < 100,000/lL, serum calcium ≥10.3 mg/dL, or total bilirubin ≥1.4 mg/dL

^a^NOTE. Primary characteristics are partially modified

### Statistical analyses

The database was analyzed using a software program (SPSS, version 23; IBM, Armonk, NY). Survival time was calculated from the start date of RT for bone metastases until death or a censored date. The survival time of patients who had been missing were censored at the last follow-up. Survival curves were constructed using the Kaplan-Meier method, and the log-rank test was used to test the difference between survival curves for each factor in a univariate analysis. A multivariate analysis was performed using the Cox proportional hazards model to identify independent predictors of death. Statistical significance was defined as *P*-values of 0.05 or less, based on two-sided tests. Missing data were excluded from the analysis.

## Results

Six hundred and sixteen patients were retrospectively reviewed in this study. The median follow-up time for survivors was 42 (range, 0–137) months, and 574 patients had died and 42 remained alive. The 3-, 6-, 12- and 24-month survival rates of the entire group were 57.7, 40.5, 23.4, and 12.2%, respectively.

The patients included 372 men and 244 women, and the median patient age was 65 (range, 26–89) years. The median scores for KPS and ECOG PS at the start of RT were 70 (range, 10–100) and 2 (range, 0–4), respectively. The laboratory data showed normal results in 35 patients (5.7%), abnormal results in 244 patients (39.6%), and critical results in 93 patients (15.1%). Lung cancer was the most common primary lesion (36.0%), followed by breast cancer (10.6%), kidney cancer (6.2%), liver cancer (6.0%), prostate cancer (5.7%), colon and rectal cancer (5.4%), stomach cancer (3.9%), and other cancers. Nodular metastases were identified in 403 patients (65.4%), and disseminated metastases ware in 96 patients (15.6%). Previous chemotherapies and multiple bone metastases were documented in 59.9 and 70.9% of patients, respectively. Of the patients in our study, 199 (32.3%) were treated by single dose fractionation schedule RT, while the remaining 417 (67.7%) underwent multiple dose fractionation schedules for bone metastasis. Upon categorization by irradiation site, 339 patients (55.0%) underwent irradiation for spinal metastases, 77 (12.5%) for extremity metastases, 161 (26.1%) for pelvic bone metastases, and 39 (6.3%) for other metastases.

Table 2 and Additional file 1 list patient and tumor characteristics, and Additional file 2 shows a summary of RT.

**Table 2:** Patient characteristics

Variable	Subgroups	Entire cohort		Validation cohort for the Katagiri’s scoring system	
		No. of Patients	%	No. of Patients	%
Gender	Men	372	60.4	225	63.2
	Women	244	39.6	131	36.8
Age	≤ 64	332	53.9	174	48.9
	≥ 65	284	46.1	182	51.1
KPS	10–60	277	45	188	52.8
	70–80	192	31	100	28.1
	90–100	131	21	66	18.5
ECOG PS	0–2	377	61	201	56.5
	3–4	226	37	155	43.5
Primary lesion	Lung cancer	222	36	163	45.8
	Breast cancer	65	11	15	4.2
	Prostate cancer	35	6	16	4.5
	Others	294	48	162	45.5
Primary characteristics	Slow	114	19	44	12.4
	Moderate	75	12	44	12.4
	Rapid	427	69	268	75.3
Visceral metastases	No	98	15.9	42	11.8
	Nodular	403	65.4	248	69.7
	Disseminated	96	15.6	66	18.5
Laboratory data	Normal	35	5.7	32	9.0
	Abnormal	244	39.6	232	65.2
	Critical	93	15.1	92	25.8
Previous chemotherapy	No	236	38.3	146	41.0
	Yes	369	59.9	210	59.0
Multiple bone metastases	No	169	27.4	86	24.2
	Yes	437	70.9	270	75.8
Katagiri score	0–3			18	5.1
	4–6			133	37.4
	7–10			205	57.6

### Predictors for survival

Sex, KPS, ECOG PS, primary lesions and characteristics, visceral metastases, laboratory data, previous chemotherapy, and Katagiri score showed significant prognostic values for survival in a univariate analysis (Table 3). There were no significant prognostic values in age (*P* = 0.604) and multiple bone metastases (*P* = 0.691).

**Table 3:** Univariate analysis for overall survival

Variable	Subgroups	Entire cohort		Validation cohort for the Katagiri’s scoring system	
		Median survival (m)	*P* value	Median survival (m)	P value
Gender	Men	8.0		3.0	
	Women	4.0	< 0.001	9.0	< 0.001
Age	≤ 64	5.0		4.0	
	≥ 65	4.0	0.604	3.0	0.529
KPS	10–60	3.0		3.0	
	70–80	5.0		4.0	
	90–100	8.0	< 0.001	8.0	0.007
ECOG PS	0–2	6.0		5.0	
	3–4	3.0	< 0.001	3.0	0.003
Primary lesion	Lung cancer	4.0		3.0	
	Breast cancer	13.0		20.0	
	Prostate cancer	8.0		8.0	
	Others	4.0	< 0.001	4.0	< 0.001
Primary characteristics	Slow	10.0		13.0	
	Moderate	8.0		8.0	
	Rapid	3.0	< 0.001	3.0	< 0.001
Visceral metastases	No	11.0		11.0	
	Nodular	4.0		4.0	
	Disseminated	2.0	< 0.001	2.0	< 0.001
Laboratory data	Normal	12.0		11.0	
	Abnormal	4.0		4.0	
	Critical	2.0	< 0.001	2.0	< 0.001
Previous chemotherapy	No	6.0		5.0	
	Yes	4.0	< 0.001	3.0	< 0.001
Multiple bone metastases	No	5.0		4.0	
	Yes	4.0	0.691	4.0	0.961
Katagiri score	0–3			27.0	
	4–6			6.0	
	7–10			2.0	< 0.001

In our multivariate analysis, sex, KPS, primary characteristics, visceral metastases, laboratory data, and previous chemotherapy maintained significance, whereas the primary legion variable lost significance (Table 4). In the multivariate analysis, sex and ECOG PS were analyzed separately.

**Table 4:** Multivariate analysis for overall survival

Variable	HR	95%CI	*P* value
Gender			
man	0.479	0.375–0.612	< 0.001
woman	1.000		
KPS			
10–60	1.796	1.340–2.408	< 0.001
70–80	1.479	1.072–2.041	0.017
90–100	1.000		
ECOG PS			
0–2	0.690	0.547–0.871	0.002
3–4	1.000		
Laboratory data			
Normal	0.312	0.203–0.480	< 0.001
Abnormal	0.697	0.541–0.900	0.006
Critical	1.000		
Primary characteristics			
Slow	0.278	0.187–0.412	< 0.001
Moderate	0.708	0.507–0.988	0.042
Rapid	1.000		
Primary lesion			
Lung			
Breast			
Prostate			
Others			NS
Visceral metastases			
No	0.428	0.279–0.655	< 0.001
Nodular	0.753	0.568–0.997	0.047
Disseminated	1.000		

*NS* not significant

### Validation of the Katagiri scoring system

Of 616 patients, we could calculate the Katagiri score in the 356 patients. The median follow-up time for survivors was 52 (range, 0–137) months. Three hundred and forty patients had died and 16 remained alive, and the 3-, 6-, 12- and 24-month survival rates of the validation cohort were 52.6, 34.9, 20.0 and 10.6%, respectively. The patient characteristics of the validation cohort are shown in Table 2. The survival rates across the different Katagiri scores demonstrated that the higher the prognostic score, the lower the survival rate (Additional file 3).

**Fig. 1:**
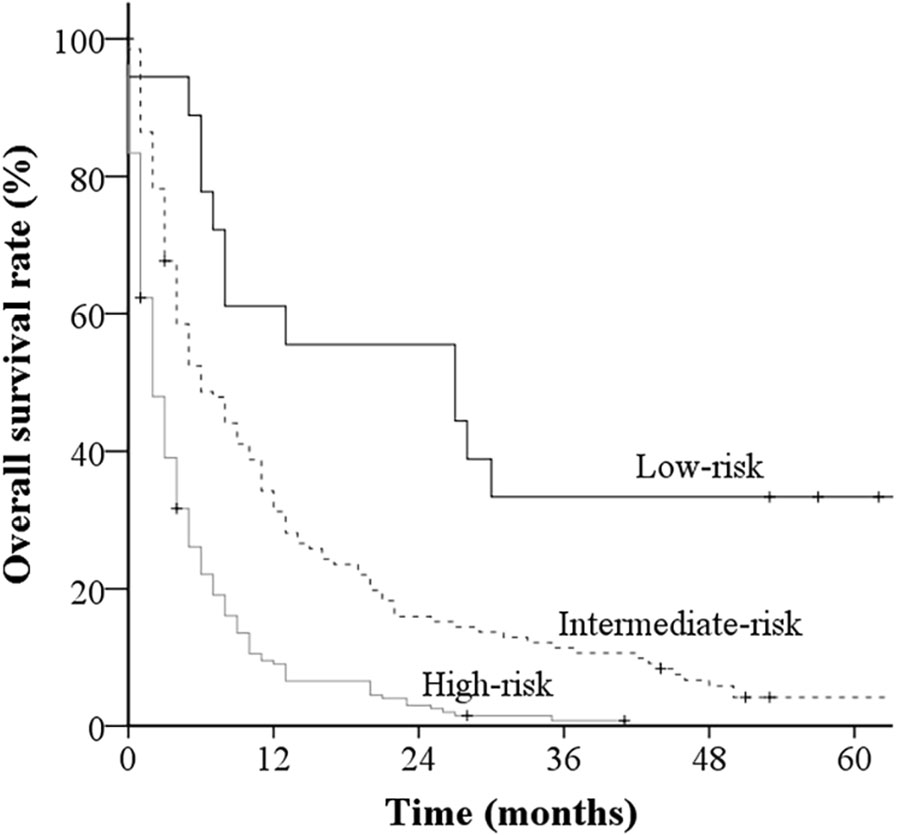
Kaplan–Meier survival curves for patients with Katagiri scores of 0–3 (low-risk group, *n* = 18), 4–6 (intermediate-risk group, *n* = 133), and 7–10 (high-risk group, *n* = 205). The rates of survival for these three groups are significantly different (*p* < 0.001)

Of the validation cohort, 18 patients were assigned to the low-risk group, 133 to the intermediate-risk group, and 205 to the high-risk group. There were significant differences in median survival times, categorized by the Katagiri scoring system, between the risk groups (low-risk group, 27 months; intermediate-risk group, 6 months; and high-risk group, 2 months) (*P* < 0.001). The 3-, 6-, 12-, and 24-month survival rates for each categorized score are as follows: low-risk group, 94.4, 77.8, 61.1, and 55.6; intermediate-risk group, 67.7, 48.7, 31.2, and 16.0; and high-risk group, 39.1, 22.1, 9.0, and 3.0, respectively (Table 5). The assigned group was highly predictive of patient outcome. These survival rates are summarized in Fig. 1.

**Table 5:** Survival rate by categorized group

Group	Total points	Median survival (m)	Survival (%)				*P* value
			3 m	6 m	12 m	24 m	
Low-risk	0–3	27	94.4	77.8	61.1	55.6	
Intermediate-risk	4–6	6	67.7	48.7	31.2	16.0	
High-risk	7–10	2	39.1	22.1	9.0	3.0	< 0.001

## Discussion

Bone metastases cause variable symptoms, such as pain, pathologic fractures, MSCC and hypercalcemia, and negatively affect the patient’s quality of life (QOL). General management of bone metastases focuses on QOL improvements. RT plays important roles in palliation of the symptoms derived from bone metastases and improving QOL. Generally, feasible RT dose-fractionation schedules for patients with bone metastases are as follows: single-fraction RT, such as 8 Gy in 1 fraction, or multi-fraction RT, such as 20 Gy in 5 fractions, 30 Gy in 10 fractions, and other dose schedules. The definition of the dose fractionation regimen mainly depends on the purpose of the RT and on patient prognosis. For the management of pain, many studies have shown that single- or multi-fraction RT were equally effective [3, 4]. Although single fractionation is convenient, it has been associated with more incidences of repeated treatments than fractionated RT [4]. It is recommended to select single-fraction RT for patients with shorter predicted survival prognoses and multi-fraction RT for other patients. For the management of MSCC, Rades et al. [5] reported 1-year in-field recurrence-free rates after radiation therapy of about 74 and 90% in short- and long-course radiation therapies, respectively, although the two regimens provided similar functional outcomes. It is recommended to use single-fraction radiation therapy for patients with poor predicted survival and longer course radiation therapy for other patients.

The Katagiri scoring system [2] allows clinical physicians to estimate the survival of patients with bone metastases, which can help select the most optimal dose-fractionation regimen for the individual patient. The scoring system includes six prognostic groups and has been developed from a large prospective series of 808 patients. However, this scoring system has not yet been validated in a RT setting. The current study included a validation group of 356 patients with bone metastases who were treated with RT. There were significant differences in median survival times among the three prognostic groups. This finding demonstrates the validity of the scoring system in an RT setting. Since the 6-month survival rates of patients in the high-risk group (Katagiri scores of 7–10) are low, these patients should be treated with single-fraction RT for pain or MSCC management. On the other hand, patients in the low-risk (Katagiri scores of 0–3) or intermediate-risk (Katagiri scores of 4–6) groups should receive multi-fraction RT, and the consideration of the patient’s preference and systemic therapy priority is also important.

Katagiri et al. [2] reported survival rates at 6, 12, and 24 months, which were categorized by the Katagiri scoring system, as follows: low-risk group, 98.1, 91.4, and 77.8; intermediate-risk group, 74.0, 49.3, and 27.6; and high-risk group, 26.9, 6.0, and 2.1, respectively. The survival rates in the current series were found to be worse than those reported by Katagiri et al., and there are several reasons for this. One reason is due to differences in the time of starting the studies. The study of Katagiri started at the time of symptomatic bone metastasis detection, while we started at the beginning of RT. Secondly, Katagiri et al. investigated patients who were treated with both surgery and RT, and in contrast, we only studied patients who underwent RT alone. 7% of the patients in the study by Katagiri et al. underwent surgery. Third, because all patients with lung cancer in this study were categorized into the rapid tumor growth cohort, and all patients with prostate and breast cancers were categorized into the slow tumor growth cohort, the rapid tumor growth group seemed better, and the slow tumor growth group seemed worse, with respect to survival.

In previous studies, the primary tumor, visceral metastases, ECOG PS, and KPS have been commonly reported as significant prognostic factors for patients who were treated with RT for bone metastases [2, 6–10]. Katagiri et al. [2] classified primary tumors into three categories, according to the median survival duration of each malignancy. Patients with cancers who had median survival times of > 20 months were classified into the slow growth group, those with median survival times of 10–20 months were classified into the moderate growth group, and those with median survival times of < 10 months were categorized into the rapid growth group. Lung cancer was categorized into two subgroups, which depended on whether the patients underwent treatment with gefitinib and/or erlotinib, and prostate and breast cancers were categorized according to their sensitivity to hormonal therapy. We simplified the categorization of lung cancer into rapid growth tumors, and breast and prostate cancers were classified as slow growth tumors, due to difficulties in retrospectively finding data. The recent progress in systemic therapies has been remarkable, especially in lung cancer, and many molecular targeted drugs, immune checkpoint inhibitors, and other therapeutics may greatly impact the classification of primary tumors. However, models that are too complex are not preferable for daily practice.

In previous studies of prognostic systems for bone metastasis, laboratory data have not been sufficiently investigated, although such data has been reported to be useful for estimating prognoses for some malignancies. CRP and serum albumin are essential factors in the Glasgow prognostic score [11], which is an inflammation-based cumulative prognostic score that is a significant prognostic tool for many malignancies. Mizumoto et al. [6] reported serum calcium level as a significant prognostic factor for patients with bone metastases who were treated with RT, and this finding is consistent with the current study. LDH and thrombocytopenia have been reported to be prognostic factors for patients with some malignancies [12–15], and hyperbilirubinemia is one of the factors of the Child-Pugh classification, which is used to assess the liver disease severity. One problem with defining a score is that a finite classification approach for such laboratory data has not been established. In the Katagiri scoring system, laboratory data are categorized based on whether they directly threaten patient life. Thus, elevated CRP, LDH, and hypoalbuminemia levels are categorized as abnormal because they do not directly threaten patient life. In contrast, hypercalcemia, thrombocytopenia, and hyperbilirubinemia can directly threaten lives. However, because this categorization is subjective, we should consider additional thorough statistical categorization approaches. Other laboratory data, such as anemia, have also been reported to be a prognostic factor for some malignancies [15, 16]. More laboratory values and categorizations need to be investigated as prognostic factors for patients with bone metastases.

Previous chemotherapy was a significant prognostic factor in our analysis and according to reports by Katagiri et al. [2] and Mizumoto et al. [6], but has not been included in the analyses of other studies. Chemotherapy itself does not worsen survival, but rather patient characteristics, such as chemo-resistance, may negatively impact survival. Moreover, RT for initial bone metastasis treatment followed by chemotherapy may impact results. Additional investigations of this factor may be required.

Multiple bone metastases were not identified as a significant prognostic factor in the current study, in contrast to the results reported by Katagiri et al. There have been inconsistencies in the significance of multiple bone metastases as a prognosis factor. This factor was determined to be a significant prognostic factor in the studies by Katagiri et al. [2], Mizumoto et al. [6] and Rades et al. [7], which were in contrast to the results reported by van der Linden et al. [8] and our results. These differences were probably caused by patient selection bias, because the current study included patients who were treated with RT for bone metastases of the entire skeleton.

Sex has been used to predict survival times in other studies. Westhoff et al. [9] reported that being male and having any primary tumor other than breast cancer were associated with higher risks of death in patients with painful bone metastases. Moreover, Nakamura et al. [17] reported that the hazard ratio for female patients with non-small-cell lung cancer (NSCLC) is 0.78, with a significant difference in survival between male and female patients.

Given the limitations of a retrospective study, the previously developed Katagiri scoring system also proved valid in patients who were treated with RT, and this system should be considered when selecting optimal dose-fractionation. Although sex, KPS, ECOG PS, characteristics of the primary site, visceral metastases, laboratory data, and previous chemotherapy were significant predictors of survival in this study, further studies are required to validate and better define the optimal prognostic factors for patients with bone metastases.

## Additional files

Additional file 1: Patient and tumor characteristics. (DOCX 20 kb)

Additional file 2: Summary of radiation therapy. (DOCX 16 kb)

Additional file 3: Katagiri score and survival rate. (DOCX 15 kb)

## Abbreviations

CRP: C-reactive protein
ECOG PS: Eastern Cooperative Oncology Group performance status
IRB: Institutional Review Board
KPS: Karnofsky performance status
LDH: Lactate dehydrogenase
MSCC: Metastatic spinal cord compression
NSCLC: Non-small-cell lung cancer
OS: Overall survival
QOL: Quality of life
RT: Radiation therapy
