# Supplementary material for: Predicting the survival of patients with bone metastases treated with radiation therapy: a validation study of the Katagiri scoring system
Source: Radiat Oncol. 2019 Jan 18;14:13. doi: 10.1186/s13014-019-1218-z (PMC6339356; doi:10.1186/s13014-019-1218-z)
Supplement: Supplementary file 1 — Patient and tumor characteristics. (DOCX 20 kb) [file 13014_2019_1218_MOESM1_ESM.docx]

Additional file 1. Patient and tumor characteristics

|  |  | Entire cohort | | |  | Validation group | | |
| --- | --- | --- | --- | --- | --- | --- | --- | --- |
|  |  | Median | No | % |  | Median | No | % |
| ECOG PS |  | 2 (0-4) |  |  |  | 2 (0-4) |  |  |
|  | 0 |  | 9 | 1.5 |  |  | 4 | 1.1 |
|  | 1 |  | 212 | 34.4 |  |  | 110 | 30.9 |
|  | 2 |  | 156 | 25.3 |  |  | 87 | 24.4 |
|  | 3 |  | 138 | 22.4 |  |  | 102 | 28.7 |
|  | 4 |  | 88 | 14.3 |  |  | 53 | 14.9 |
| KPS |  | 70 (10-100) |  |  |  | 60 (10-100) |  |  |
|  | 100 |  | 6 | 1.0 |  |  | 4 | 1.1 |
|  | 90 |  | 125 | 20.3 |  |  | 62 | 17.4 |
|  | 80 |  | 92 | 14.9 |  |  | 43 | 12.1 |
|  | 70 |  | 100 | 16.2 |  |  | 57 | 16.0 |
|  | 60 |  | 88 | 14.3 |  |  | 57 | 16.0 |
|  | 50 |  | 79 | 12.8 |  |  | 59 | 16.6 |
|  | 40 |  | 78 | 12.7 |  |  | 46 | 12.9 |
|  | 30 |  | 24 | 3.9 |  |  | 19 | 5.3 |
|  | 20 |  | 7 | 1.1 |  |  | 6 | 1.7 |
|  | 10 |  | 1 | 0.2 |  |  | 1 | 0.3 |
|  | 0 |  | 0 | 0.0 |  |  | 0 | 0.0 |
| Distribution of primary tumor | | |  |  |  |  |  |  |
|  | Lung |  | 222 | 36.0 |  |  | 163 | 45.8 |
|  | Breast |  | 65 | 10.6 |  |  | 15 | 4.2 |
|  | Kidney |  | 38 | 6.2 |  |  | 23 | 6.5 |
|  | Liver |  | 37 | 6.0 |  |  | 12 | 3.4 |
|  | Prostate |  | 35 | 5.7 |  |  | 16 | 4.5 |
|  | Colon |  | 33 | 5.4 |  |  | 15 | 4.2 |
|  | Gastric |  | 24 | 3.9 |  |  | 13 | 3.7 |
|  | Unknown |  | 23 | 3.7 |  |  | 20 | 5.6 |
|  | Urinary tract |  | 20 | 3.2 |  |  | 5 | 1.4 |
|  | Head and Neck | | 19 | 3.1 |  |  | 11 | 3.1 |
|  | Esophagus |  | 19 | 3.1 |  |  | 10 | 2.8 |
|  | Endometrioid | | 12 | 1.9 |  |  | 6 | 1.7 |
|  | Sarcoma |  | 11 | 1.8 |  |  | 6 | 1.7 |
|  | Biliary tract |  | 8 | 1.3 |  |  | 5 | 1.4 |
|  | Cervix |  | 8 | 1.3 |  |  | 3 | 0.8 |
|  | Melanoma |  | 7 | 1.1 |  |  | 4 | 1.1 |
|  | Myeloma |  | 5 | 0.8 |  |  | 5 | 1.4 |
|  | Lymphoma |  | 4 | 0.6 |  |  | 3 | 0.8 |
|  | Ovary |  | 4 | 0.6 |  |  | 3 | 0.8 |
|  | Pancreus |  | 4 | 0.6 |  |  | 4 | 1.1 |
|  | Thyroid |  | 3 | 0.5 |  |  | 2 | 0.6 |
|  | Others |  | 15 | 2.4 |  |  | 12 | 3.4 |
| Laboratory data | |  |  |  |  |  |  |  |
| CRP |  | 1.5 (0-38.6) |  |  |  | 1.8 (0-38.6) |  |  |
|  | <0.4 mg/dL |  | 109 | 17.7 |  |  | 64 | 18.0 |
|  | ≥ 0.4 mg/dL |  | 385 | 62.5 |  |  | 292 | 82.0 |
| LDH |  | 252(114-11440) |  |  |  | 264(114-11440) |  |  |
|  | < 250 IU/L |  | 263 | 42.7 |  |  | 163 | 45.8 |
|  | ≥ 250 IU/L |  | 268 | 43.5 |  |  | 193 | 54.2 |
| Alb |  | 3.6 (1.6-4.9) |  |  |  | 3.6 (1.6-4.7) |  |  |
|  | <3.7 g/dL |  | 268 | 43.5 |  |  | 195 | 54.8 |
|  | ≥3.7 g/dL |  | 245 | 39.8 |  |  | 161 | 45.2 |
| Ca |  | 9.5(8.1-15) |  |  |  | 9.5(8.1-14.4) |  |  |
|  | <10.3 mg/dL |  | 388 | 63.0 |  |  | 291 | 81.7 |
|  | ≥10.3 mg/dL |  | 74 | 12.0 |  |  | 65 | 18.3 |
| Plt |  | 23(1.4-113.9) |  |  |  | 23.6(1.4-113.9) |  |  |
|  | <100,000/lL |  | 38 | 6.2 |  |  | 19 | 5.3 |
|  | ≥100,000/lL |  | 514 | 83.4 |  |  | 337 | 94.7 |
| T-Bil |  | 0.5 (0.1-11.6) |  |  |  | 0.5(0.1-11.6) |  |  |
|  | <1.4 mg/dL |  | 518 | 84.1 |  |  | 337 | 94.7 |
|  | ≥1.4 mg/dL |  | 28 | 4.5 |  |  | 19 | 5.3 |
