# Supplementary material for: Predicting the survival of patients with bone metastases treated with radiation therapy: a validation study of the Katagiri scoring system
Source: Radiat Oncol. 2019 Jan 18;14:13. doi: 10.1186/s13014-019-1218-z (PMC6339356; doi:10.1186/s13014-019-1218-z)
Supplement: Supplementary file 2 — Summary of radiation therapy. (DOCX 16 kb) [file 13014_2019_1218_MOESM2_ESM.docx]

Additional file 2. Summary of radiation therapy

|  |  | Entire cohort | | |  | Validation group | | |
| --- | --- | --- | --- | --- | --- | --- | --- | --- |
|  |  | Median | No | % |  | Median | No | % |
| Distribution of irradiated bone metastases | | | |  |  |  |  |  |
|  | Vertebral bone | | 339 | 55.0 |  |  | 188 | 52.8 |
|  | Pelvic bone |  | 161 | 26.1 |  |  | 97 | 27.2 |
|  | Limbs |  | 77 | 12.5 |  |  | 50 | 14.0 |
|  | Others |  | 39 | 6.3 |  |  | 21 | 5.9 |
| Planed dose-fractionation | | 30 (8-56) |  |  |  | 30 (8-56) |  |  |
|  | 8Gy/1fr |  | 198 | 32.1 |  |  | 128 | 36.0 |
|  | 20Gy/5fr |  | 70 | 11.4 |  |  | 41 | 11.5 |
|  | 30Gy/10fr |  | 289 | 46.9 |  |  | 163 | 45.8 |
|  | Others |  | 59 | 9.6 |  |  | 24 | 6.7 |
| Irradiated dose-fractionation | | 30 (0-56) |  |  |  | 21 (6-56) |  |  |
|  | 8Gy/1fr |  | 199 | 32.3 |  |  | 129 | 36.2 |
|  | 20Gy/5fr |  | 66 | 10.7 |  |  | 40 | 11.2 |
|  | 30Gy/10fr |  | 274 | 44.5 |  |  | 152 | 42.7 |
|  | Others |  | 77 | 12.5 |  |  | 35 | 9.8 |
| Irradiation site | |  |  |  |  |  |  |  |
|  | Spinal metastases | | 339 | 55.0 |  |  | 188 | 52.8 |
|  | Extremity metastases | | 77 | 12.5 |  |  | 50 | 14.0 |
|  | pelvic bone metastases | | 161 | 26.1 |  |  | 97 | 27.2 |
|  | Others |  | 39 | 6.3 |  |  | 21 | 5.9 |
