# Supplementary material for: Predicting the survival of patients with bone metastases treated with radiation therapy: a validation study of the Katagiri scoring system
Source: Radiat Oncol. 2019 Jan 18;14:13. doi: 10.1186/s13014-019-1218-z (PMC6339356; doi:10.1186/s13014-019-1218-z)
Supplement: Supplementary file 3 — Katagiri score and survival rate. (DOCX 15 kb) [file 13014_2019_1218_MOESM3_ESM.docx]

Additional file 3. Katagiri score and survival rate

| Katagiri score | No | % | Median survival (m) | Survival (%) | | | |
| --- | --- | --- | --- | --- | --- | --- | --- |
|  |  |  |  | 3m | 6m | 12m | 24m |
| 0 | 0 | 0.0 |  |  |  |  |  |
| 1 | 0 | 0.0 |  |  |  |  |  |
| 2 | 8 | 2.2 | 30 | 87.5 | 87.5 | 87.5 | 75.0 |
| 3 | 10 | 2.8 | 7 | 90.0 | 60.0 | 40.0 | 40.0 |
| 4 | 26 | 7.3 | 20 | 76.9 | 73.1 | 65.4 | 46.2 |
| 5 | 38 | 10.7 | 8 | 81.6 | 52.6 | 31.6 | 10.5 |
| 6 | 69 | 19.4 | 4 | 56.5 | 37.2 | 17.8 | 7.4 |
| 7 | 107 | 30.1 | 4 | 55.7 | 31.8 | 13.5 | 4.8 |
| 8 | 64 | 18.0 | 2 | 27.3 | 16.1 | 6.4 | 1.6 |
| 9 | 31 | 8.7 | 1 | 9.7 | 3.2 | 0.0 | 0.0 |
| 10 | 3 | 0.8 | 1 | 0.0 | 0.0 | 0.0 | 0.0 |
